# Factors and Predictors of Health Related Quality of Life of the General Population of Pakistan

**DOI:** 10.3389/fpubh.2022.819088

**Published:** 2022-08-17

**Authors:** Aqeel Nasim, Noman Ul Haq, Sohail Riaz, Sumaira Irum Khan, Fazli Khuda, Muhammad Faraz Sipra, Bazil Tariq, Maria Tahir, Muhammad Saood, Riffat Yasmin, Kiran Manzoor, Muhammad Zeeshan Danish

**Affiliations:** ^1^Faculty of Pharmacy and Health Sciences, University of Balochistan, Quetta, Pakistan; ^2^Faculty of Pharmacy, Capital University of Science and Technology, Islamabad, Pakistan; ^3^Department of Pharmacy, Mirpur University of Science and Technology, Mirpur, Pakistan; ^4^Department of Pharmacy, University of Peshawar, Peshawar, Pakistan; ^5^Department of Pathology, Shifa College of Medicine, Shifa Tameer-e-Millat University, Islamabad, Pakistan; ^6^Department of Physiology, Army Medical College, Rawalpindi, Pakistan; ^7^Department of Pharmacy, Sardar Bahadur Khan Women University, Quetta, Pakistan; ^8^Faculty of Management Sciences, Balochistan University of Information Technology, Engineering and Management Sciences, Quetta, Pakistan; ^9^Punjab University College of Pharmacy, University of the Punjab, Lahore, Pakistan

**Keywords:** EQ5D 3L, HRQOL, norms, quality of life (QoL), health

## Abstract

**Background and Objective:**

The standards of living, improvement in public health, and medical care in Pakistan are increasing day by day, health-related quality of life (HRQoL) has been increasingly acknowledged in various patient's reported outcomes in Pakistan. However, a large-scale general population-based study on assessing HQRoL in Pakistan was not conducted. Therefore, this study aimed to evaluate HRQoL for the general Pakistani population.

**Material and Methods:**

A cross-sectional study with a population sample (*n* = 16,672) was selected from all Pakistan provinces using a stratified sampling approach. The EQ-5D-3L tool was used to measure the HRQoL of the general population of Pakistan. The descriptive and inferential statistics have been done by using SPSS version 20.

**Results:**

Overall, 121 health states were reported in this study. EQ-5D index and EQ-VAS scores were 0.74 ± 0.32 and 0.75 ± 0.25, respectively. The percentage of people responding to any problems increased with age. Males have better health as compared to females in all age groups. All demographics were significantly associated (*P* < 0.01) with the mean EQ5D index and VAS scores except residence (*p* > 0.05). The regression model reported that age was the best predictor of the EQ-5D index scores after adjusting for the covariates (beta = 0.19; *p* < 0.001). This study provides Pakistani population HRQoL data measured by the EQ-5D tool, based on a national representative sample.

**Conclusion:**

The current study concluded that Age, City, Gender, Education, Occupation, Residence, and House occupancy are significantly affecting HRQOL. The socioeconomically deprived groups and females have inferior health status than more advantaged. The trends detected in high-income nations were usually similar to Pakistan.

## Introduction

World Health Organization (WHO) defined health as “a state of complete physical, mental, and social wellbeing and not merely the absence of disease or infirmity” (1). As discussed as a matter of wellbeing, it reflects the quality of life (QoL). The QoL is a multidimensional construct primarily based on a person's subjective appraisal of physical, functional, emotional, and social wellbeing. While health is one of the significant aspects of the overall quality of life (2). When considered an element of quality of life, health is the best aspect that is generally considered to fall within healthcare professionals' scope or that is probably the goal of medical intervention; therefore, the term “health-related quality of life (HRQoL)” is preferred over QoL. HRQoL, On the individual level includes physical and mental health perceptions (e.g., energy level, mood) and their correlates—including health risks and conditions, functional status, social support, and socioeconomic status. On the community level, HRQoL includes community-level resources, conditions, policies, and practices that influence a population's health perceptions and functional status (3).

There are two basic approaches to measure health-related quality of life (HRQoL): general tools that summarize the health-related quality of life and specific tools that focus on issues related to individual disease states, patient groups, or functional areas. Standard tools generate utility measures for health profiles and HRQoL (4). However, tools used in the measurements of both specific and population HRQoL are either generic, i.e., not specifically designed for patients with a particular disease, or they may be specific for a specific disease or condition but not applicable to the general population (3).

Measures of HRQoL are considered to provide measurable results for health interventions. They are an integral component of evidence-based public health policy and point to health's ultimate goal for all (5). A national HRQOL standard provides policymakers with a common set of criteria for assessing public health improvement and can give a general indicator of care quality (6).

Pakistan is an agricultural country situated northwestern in the Asian subcontinent having an area of 796,096 sq. K.M. with a mean G.D.P. per capita in 2016 of $1,428, while Pakistan spends 0.9pc of its G.D.P. on health (7). Pakistan consists of four provinces: Punjab, Sindh, Balochistan, and Khyber Pakhtunkhwa (K.P.K.). In addition, federally Administrative Tribal Areas and the Gilgit Baltistan region are federally administered areas (8). Since the 1990's, three health surveys have been conducted in Pakistan. The last one was completed in 2013, conferring to Pakistan Demographic and Health Survey (PDHS) to deliver data for assessing Pakistan's people and health conditions. This survey covered parameters belonging to women's and children's health state, Communicable disease, i.e., H.I.V. (AIDS), allowing policymakers to make plans accordingly (8). Unfortunately, these investigations do not gather HRQoL data in the general population of Pakistan.

The EQ-5D has been used in Pakistan to assess HRQoL among individuals with a disease state. The outcomes of a clinical intervention obtained by the patient, i.e., patient-reported outcomes (P.R.O.s), seem to be of more importance in the future than any other outcomes like clinical, physiological, or caregiver-reported (9). This PRO assesses HRQoL by using EQ-5D in hepatitis patients (10, 11), hemodialysis patients (12), patients with mental illness (13), among pregnant women (14), CHD patients (15, 16), End-stage renal failure (ESRF) patients (17) and patients with thyroid diseases (18).

These studies do not adequately reflect the general population's health status and the nature of diseases transformed from acute to chronic diseases and community life with ill health. Non-communicable conditions, including cardiovascular diseases, cancers, respiratory diseases, diabetes, mental complaints, and injuries, have become the main reasons for morbidity and mortality in Pakistan (19), The burden in Pakistan (20). Therefore, the measurement of health status in Pakistan is highly recommended.

The population norms for the EQ-5D by socio-demographic are presented from other countries such as Australia (21), Brazil (22), U.K. (23), Sri Lanka (24), U.S. (25), Danish (26), and China (27). These Norms can be used to compare the health status of specific groups, i.e., disease state, with that of the general population. In addition, these Population norms are an essential reference point for assessing health programs and policies (28).

No study has ever been conducted in Pakistan by using any tool to assess the HRQoL of the general population. However, it is a need of time to have set of values of HRQoL of Pakistan itself as the Pakistani population norms are different from that of other developed countries. Therefore, this study aimed to evaluate HRQoL for the general Pakistani population and to determine the factors affecting HRQoL of Pakistani population.

## Methods

### Study Design and Data Collection

A Cross-sectional descriptive study was conducted on national level hence all major cities of Pakistan were included to recruit the sample which would represent the set of values of HRQoL for Pakistan. The data were collected with an EQ-5D-3L health state valuation study in Pakistan from a population sample of 16,672 persons, drawn from each province's capital and most populous Pakistan cities. They included; Federal territories, which include Islamabad and Rawalpindi; Islamabad is the Capital of Pakistan. However, based on the I.C.T. division Rawalpindi is now included in Islamabad territory. Sindh is the southeastern province of Pakistan of the third largest according to the area in Pakistan. Based on Pakistan's economy, Sindh has Pakistan's 2nd principal economy. Sindh comprises more than seventy cities, the most populous are Karachi, Hyderabad, and Sukkur, from which data were derived. Punjab is the most populous province of Pakistan and the second-largest according to the area in Pakistan. Based on the Pakistan economy, Punjab is the most industrialized province in Pakistan, with 24% G.D.P. More than hundreds of cities are there; the most populous are Lahore, Faisalabad, Multan, Gujranwala, and Sargodha. On this basis, sample data from Punjab was taken from Lahore, Faisalabad, Multan, and Sargodha.

K.P.K. in the northwestern province of Pakistan, previously known as North-West Frontier Province (NWFP). The capital of this province is Peshawar, K.P.K. has more than 40 cities, and data collection was done from Peshawar and Swat. According to the area, Balochistan is the southwestern province of Pakistan, the largest province of Pakistan. Its capital is Quetta city. Based on locality, the dominant locality was Baloch and Pashtun; on this basis, samples were taken from Quetta, Loralai, and Sibi. Azad Jammu and Kashmir were known as AJ&K, are a self-governing administrative division of Pakistan. Data was collected from Muzaffarabad. Gilgit Baltistan, previously known as the northern area of Pakistan, is the administrative territory. The capital of this province is Gilgit. Based on this, data was collected from Gilgit.

### Participants

#### Inclusion Criteria

All the general population of Pakistan who was not diagnosed with any disease was included in this study. In addition, having age 18 years who know about Urdu and can read are included who agreed to participate in the study.

#### Exclusion Criteria

Those who have any confirmed clinical condition or chronic disease will be excluded. In addition, those who do not know Urdu, do not agree to participate, pregnant females, refugees from other countries, psychological conditions were excluded.

### Sampling Procedure

Samples were derived from every city by stratified sampling technique; in this technique, the entire population (Country) into different subgroups or strata (populous cities), then randomly selected the final subjects proportionally from the various strata.; there were other data collection methods like an interview, self-reporting, which can be used as EQ-5D is a self-reported questionnaire. The interviewers from various cities were trained and given all information necessary before taking data. Both methods collected the data face to face and by sharing and collecting Questionnaires back by trained interviewers.

### Sample Size

The Raosoft sample size calculator considers the sample size with a confidence interval of 95% and a 5% margin of error. According to Raosoft, 385 samples from each city were intended to gather; the sample size does not vary for populations higher than 20,000, according to Raosoft (29). so for more significant sample size, we got 1,155 samples for this study to satisfy the target audience. The response will be monitored better; we added dropout 30%, so sample size after dropout became 1,155 + 30% = 345.5; therefore, 1,501 samples were derived.

### Study Tool

European Quality of Life scale EQ-5D-3L was used to measure HRQoL. EQ-5D is a standardized generic HRQoL instrument developed by the EuroQol Group (30). EQ-5D consists of five domains (i.e., mobility, self-care, usual activities, pain/discomfort, and anxiety/depression). These can be further categorized into three levels of severity (no problems, some or moderate problems, and extreme situations). Two hundred and twenty-six different health responses can be achieved, describing the health status of respondents. VAS (visual analog scale) is the other portion of EQ-5D; this scale ranged from 0 (worst imaginable health state) – 100 (the best possible health state). The participants were requested to point out “which point best fits your health state today.” The interviewer from various cities was trained and given all information necessary before taking data. Both methods collected the data face to face and by sharing and collecting Questionnaires back by trained interviewers. This study was registered with EuroQoL. The internal consistency and validity of the questionnaire was ensured (the Cronbach's alpha value being 0.65 for the instrument used in the study) (30).

### Ethical Approval

The Faculty of Pharmacy and Health Sciences University of Baluchistan Quetta's ethical committee has approved the study as per the guidelines of the National bioethics committee of Pakistan (31). The participants provided written informed consent before the commencement of data collection.

### Data Analysis

All analyses were done using SPSSv20. The people who reported no problems, moderate problems and severe problems for each dimension were calculated. The percentage of people reporting any issue in each dimension was calculated for the total sample and stratified by various demographic variables. Chi-square tests were used to determine the significance between groups in categorical variables. Besides these, non-parametric tests (Mann-Whitney U tests for two categories or Kruskal-Wallis tests for more than two categories) to examine differences in the EQ-5D-3L index score of the respondents because the distribution of data was skewed. To better understand the respondents' health problems, the percentage of people reporting any problem (some problems or extreme problems) in each dimension was calculated, and x^2^ tests were performed to determine the statistical significance of the difference between groups in the percentage of reported problems.

*Binary logistic regression* was then used to investigate the association between having any problem in each dimension and socio-demographic variables. The significant variables were considered the main effects, and subsequent interactions were tested in a logistic regression model. Logistic regression models were developed with the five health dimensions as dependent variables (0 = no problem, 1 = some/extreme problems). The results are presented as odds ratios (OR).

*Linear regression* was used to investigate the EQ-5D Index score because it is another dependent variable, so Linear regression was performed. Again, different socio-demographic covariates were acting as predictors and the index as the dependent variable. In this case, the weight of each covariate is the beta-coefficient.

## Results

### Demographic Characteristics

The demographic data have been presented in Table 1. Among the population selected for the study, the mean age was 29.22 years while the minimum age was 18, and the maximum was 67; among them (*n* = 9,014, 54.1%) were males while the females were (*n* = 7,658, 45.9%). Marital status showed that maximum respondents (*n* = 9,836, 59.0%) were single or unmarried. Study participants with various education maximum (*n* = 4,629, 27.8%) had a bachelor's education degree. Occupation result highlights that mainstream (*n* = 3,088, 18.5%) occupation among respondents was a Private Job. The majority of respondents (*n* = 4,418, 26.5%) had no income. Maximum study participants (*n* = 1,426, 85.5%) belonged to the Urban area and having their own house said by maximum respondents (*n* = 13,484, 80.9%). Study participants were from various cities of which main cities highlighted as Islamabad (*n* = 1,313, 7.9%), Karachi (*n* = 1,215, 7.3%), Quetta (*n* = 1,507, 9.0%), Peshawar (*n* = 1,237, 7.4%), Lahore (*n* = 1,270, 7.6%), AJK (*n* = 445, 2.7%), Gilgit Baltistan (*n* = 445, 2.7%).

**Table 1:** Demographic characteristics.

**Description**	**Frequency (*N* = 16,672)**	**Percentage**
**Age** (29.22 ± 11.63)		
18–27 Years 28–37 Years 38–47 Years 48–57 Years 58–67 Years	10,107 3,221 1,723 814 807	60.6 19.3 10.3 4.9 4.8
**Gender**		
Male Female	9,014 7,658	54.1 45.9
**Marital Status**		
Single Married Widowed	9,849 6,643 180	59.1 39.8 1.1
**Education**		
No Education Primary Middle Matric Intermediate Bachelors Graduate Masters M.Phil./PhD	900 324 443 1,782 3,621 4,629 1,881 2,909 183	5.4 1.9 2.7 10.7 21.7 27.8 11.3 17.4 1.1
**Occupation**		
Student Own business Government job Private job Engineer Doctor Labor House wife Jobless None Teacher	2643 1169 2111 3088 484 652 543 1033 1214 1959 1776	15.9 7.0 12.7 18.5 2.9 3.9 3.3 6.2 7.3 11.8 10.7
**Income**		
No income 1–5,000 PKR 5,001–10,000 PKR 10,001–20,000 PKR 20,001–30,000 PKR 30,001–40,000 PKR 40,001–50,000 PKR More than 50,000 PKR Not disclosed	4,418 961 819 2,706 3,057 1,120 773 700 2,118	26.5 5.8 4.9 16.2 18.3 6.7 4.6 4.2 12.7
**Residence**		
Urban Rural	14,262 2,410	85.5 14.5
**House occupancy**		
Own Rent	13,484 3,188	80.9 19.1
**City**		
Islamabad Rawalpindi Karachi Hyderabad Sukkur Quetta	1,313 1,269 1,215 1,248 606 1,507	7.9 7.6 7.3 7.5 3.6 9.0
Sibi Loralai Peshawar Sawat Lahore Sargodha Faisalabad Multan A.J.K. Gilgit Baltistan	1,198 1,187 1,237 734 1270 1330 1232 408 445 473	7.2 7.1 7.4 4.4 7.6 8.0 7.4 2.4 2.7 2.8

*1 PKR = 0.0071$*.

### EQ-5D Dimensions

Twelve thousand three hundred and four (73.8%) respondents showed no problem in the first domain (Mobility), 13,933 (83.6%) specified no problem in the second domain (self-care), 100,981 (65.9%) shown no problems in the third domain (Usual Work), 9,950 (59.7%) indicated no pain and discomfort in the fourth domain (Pain and Discomfort) and 9,387 (56.3%) reported not anxious or depressed in the fifth domain (Anxiety and Depression) as shown in Table 2.

**Table 2:** EQ-5D dimensions.

**Description**	**Frequency (*N* = 16,672)**	**Percentage**
**Mobility**		
No problems in walking about Some problems in walking about Confined to bed	12,304 3,603 765	73.8 21.6 4.6
**Self-care**		
No problems with self-care Some problems washing or dressing myself Unable to wash or dress myself	13,933 2,168 571	83.6 13.0 3.4
**Usual activities**		
No problems with performing my usual activities Some problems with performing my usual activities Unable to perform my usual activities	10,981 5,101 590	65.9 30.6 3.5
**Pain and discomfort**		
No pain or discomfort Moderate pain or discomfort Extreme pain or discomfort	9,950 5,931 791	59.7 35.6 4.7
**Anxiety / depression**		
Not anxious or depressed Moderately anxious or depressed Extremely anxious or depressed	9,387 6,144 1,141	56.3 36.9 6.8

### EQ-5D Health States

A total of 121 health conditions were obtained. EQ-5D descriptive score and EQ-VAS scores were 0.74 ± 0.32 and 0.75 ± 0.25, respectively. The ten furthermost frequently detected self-reported health states on the 3L descriptive systems are shown in Table 3. The cumulative frequency of the top 10 most frequently observed health states was under 74.4 %. The remaining 25.6 % of states were distributed over 111 health states.

**Table 3:** The EQ-5D health states.

**S. No**.	**Domains**	**Frequency** **(*N* = 16,672)**	**Percentage**
1.	11111	6,263	37.6
2.	11112	1,286	7.7
3.	11122	1,009	6.1
4.	11121	706	4.2
5.	22222	621	3.7
6.	11222	577	3.5
7.	11211	542	3.3
8.	21222	527	3.2
9.	11212	455	2.7
10.	11221	359	2.2
**Total**		**12,345**	**74.2%**

Six thousand two hundred and sixty-three (37.6%) reported no problem in the (11,111); first, second, third, fourth, and fifth domain, followed by 1,286 (7.7%) reported (11,112); no problem in the first, second, third and fourth domain whereas some problem in fifth domain. The incidence of the worst probable health (33,333) state was the lowest (0.9 %).

### EQ-5D Dimensions and Health-Related Quality of Life by Age, Gender and House Occupancy, Marital Status, and Income

Health status decrease with an increase in age moderate and severe problems stated in each EQ-5D scope. The mean VAS score is reduced with age, as showed in Table 4A. The mean VAS score was 74.04, Level 1 (No Problems) was reported maximum in early age groups for all EQ-5D scopes. In contrast, Level 2 and Level 3 (moderate problems) and (severe problems), respectively, were seen in older peoples in all EQ-5D 3L Dimensions.

**Table 4A:** Percentage of respondents reporting EQ-5D dimensions and HRQOL by age, gender and house occupancy, and marital status.

**EQ-5D Dimensions**		**Age**						**Gender**			**House Occupancy**			**Marital Status**			
		**18–27** **Years**	**28–37** **Years**	**38–47** **Years**	**48–57** **Years**	**58–67** **Years**	***P*–Value**	**Male**	**Female**	***P*–Value**	**Own**	**Rent**	***P*–Value**	**Single**	**Married**	**Widowed**	***P*–Value**
Mobility	Level 1	80.2%	75.7%	59.3%	57.2%	33.6%	**0.001**	76.6%	70.5%	**0.001**	75.7%	65.8%	**0.001**	64.3%	34.7%	1.0%	**0.001**
	Level 2	15.8%	21.9%	33.8%	38.7%	49.8%		18.9%	24.8%		20.5%	26.3%		42.3%	56.3%	1.4%	
	Level 3	4.0%	2.5%	6.8%	4.1%	16.6%		4.5%	4.7%		3.8%	7.8%		53.6%	44.8%	1.6%	
Self-care	Level 1	85.8%	84.8%	83.2%	78.1%	57.5%	**0.001**	83.3%	83.9%	**0.343**	85.1%	76.9%	**0.001**	61.1%	38.0%	1.0%	**0.001**
	Level 2	11.8%	12.7%	9.6%	16.5%	33.1%		13.1%	12.9%		11.9%	17.8%		49.7%	48.9%	1.4%	
	Level 3	2.4%	2.5%	7.1%	5.4%	9.4%		3.6%	3.2%		3.0%	5.3%		46.2%	51.7%	2.1%	
Usual Activities	Level 1	71.3%	64.4%	53.8%	60.6%	35.3%	**0.001**	64.9%	67.0%	**0.002**	67.0%	61.1%	**0.001**	70.3%	59.9%	48.2%	**0.001**
	Level 2	25.7%	34.5%	41.8%	35.9%	47.6%		31.7%	29.3%		30.1%	32.8%		26.7%	36.2%	37.8%	
	Level 3	3.1%	1.1%	4.4%	3.6%	17.1%		3.4%	3.7%		3.0%	6.0%		3.1%	4.0%	14.0%	
Pain / discomfort	Level 1	68.6%	55.7%	44.2%	33.8%	23.4%	**0.001**	63.9%	54.7%	**0.001**	61.3%	52.9%	**0.001**	67.6%	48.7%	35.2%	**0.001**
	Level 2	28.5%	39.7%	47.4%	60.4%	57.7%		31.9%	40.0%		34.6%	39.7%		29.3%	44.7%	44.6%	
	Level 3	2.9%	4.7%	8.4%	5.8%	18.8%		4.2%	5.4%		4.1%	7.4%		3.1%	6.7%	20.2%	
Anxiety / depression	Level 1	60.5%	52.2%	47.5%	40.8%	54.4%	**0.001**	58.7%	53.5%	**0.001**	58.1%	48.9%	**0.001**	60.9%	50.0%	40.4%	**0.001**
	Level 2	34.4%	41.8%	43.2%	43.1%	27.8%		34.7%	39.4%		36.7%	37.6%		33.8%	41.5%	34.7%	
	Level 3	5.1%	6.0%	9.3%	16.1%	17.8%		6.7%	7.1%		5.3%	13.5%		5.4%	8.5%	24.9%	
EQ-5D VAS Score (Mean)		77.46	73.35	67.15	61.73	54.36	—	76.32	71.36	—	75.23	69.01	—	77.86	68.73	62.11	—

*Level 1, No Problem; Level 2, Moderate Problem; Level 3, Severe Problem*.

Comparatively better mean EQ-5D VAS score was seen in Males (76.32) as of females (71.36). EQ-5D dimensions showed that Level 1 (No Problems) were reported mainly by males in Mobility, Pain/Discomfort, and Anxiety/Depression. In contrast, females reported Level 1 (No Problems) in usual activates. Self-care was balanced among gender in terms of no problems. Females significantly reported level 2 and Level 3 (some Problems) and (severe Problems) respectively in Pain/Discomfort and Anxiety/Depression. Overall, people who are single or unmarried had better mean EQ-5D VAS (77.86) than those who were married or widowed (68.73) and (62.11), respectively. Unequal EQ-5D VAS saw among House occupancy groups, high EQ-5D VAS (75.23) seen in respondents having their own house compared to those who live on rent.

High EQ-5D VAS reported in income group whose monthly income was 20,001–30,000 RS followed by income more than 50,000 RS, as showed in Table 4B. Moderate and severe problems are reported in the EQ-5D dimension associated with Usual Activities, Pain. /Discomfort and Anxiety/Depression increased, and the mean VAS score decreased with most minor income groups, i.e., 1–5,000 RS, as showed in Table 4B.

**Table 4B:** Percentage of respondents reporting EQ-5D dimensions and HRQOL by income.

**EQ-5D Dimensions**		**Income (P.K.R.)**									
		**No Income**	**1–5,000**	**5,001–10,000**	**10,001–20,000**	**20,001–30,000**	**30,001–40,000**	**40,001–50,000**	**>50,000**	**Not disclosed**	***P*–Value**
Mobility	Level 1	74.8%	78.4%	72.8%	78.2%	72.4%	72.7%	62.4%	74.6%	74.8%	**0.001**
	Level 2	20.9%	18.2%	24.4%	16.9%	22.1%	21.7%	34.2%	20.3%	20.9%	
	Level 3	4.3%	3.4%	2.8%	4.9%	5.5%	5.6%	3.5%	5.1%	4.3%	
Self-Care	Level 1	85.5%	85.0%	86.3%	87.1%	83.3%	78.4%	76.2%	94.7%	85.5%	**0.001**
	Level 2	11.2%	15.0%	13.2%	9.6%	13.2%	12.6%	19.1%	3.3%	11.2%	
	Level 3	3.3%	0.0%	0.5%	3.3%	3.5%	9.0%	4.7%	2.0%	3.3%	
Usual	Level 1	69.3%	71.5%	60.8%	71.0%	65.0%	63.4%	53.0%	65.4%	69.3%	**0.001**
Activities	Level 2	27.3%	23.8%	38.5%	26.3%	29.8%	34.1%	44.2%	33.4%	27.3%	
	Level 3	3.4%	4.7%	0.7%	2.7%	5.1%	2.5%	2.7%	1.1%	3.4%	
Pain / Discomfort	Level 1 Level 2 Level 3	58.3% 35.8% 5.9%	73.2% 25.9% 0.9%	52.3% 40.9% 6.8%	61.2% 35.4% 3.4%	64.9% 30.2% 4.9%	52.4% 39.1% 8.5%	56.5% 39.2% 4.3%	60.0% 37.6% 2.4%	58.3% 35.8% 5.9%	**0.001**
Anxiety / Depression	Level 1 Level 2 Level 3	55.7% 36.6% 7.7%	68.0% 26.5% 5.5%	46.8% 42.6% 10.6%	57.6% 37.6% 4.8%	57.2% 34.8% 8.0%	58.1% 38.6% 3.3%	55.6% 40.0% 4.4%	71.3% 20.7% 8.0%	55.7% 36.6% 7.7%	**0.001**
VAS Score (Mean)		**73.14**	**73.09**	**66.69**	**72.95**	**76.32**	**74.56**	**72.92**	**76.02**	**75.90**	—

*Level 1, No Problem; Level 2, Moderate Problem; Level 3, Severe Problem*.

### Mean E.Q. 5D VAS vs. Age Groups in Terms of Gender

**Figure 1:**
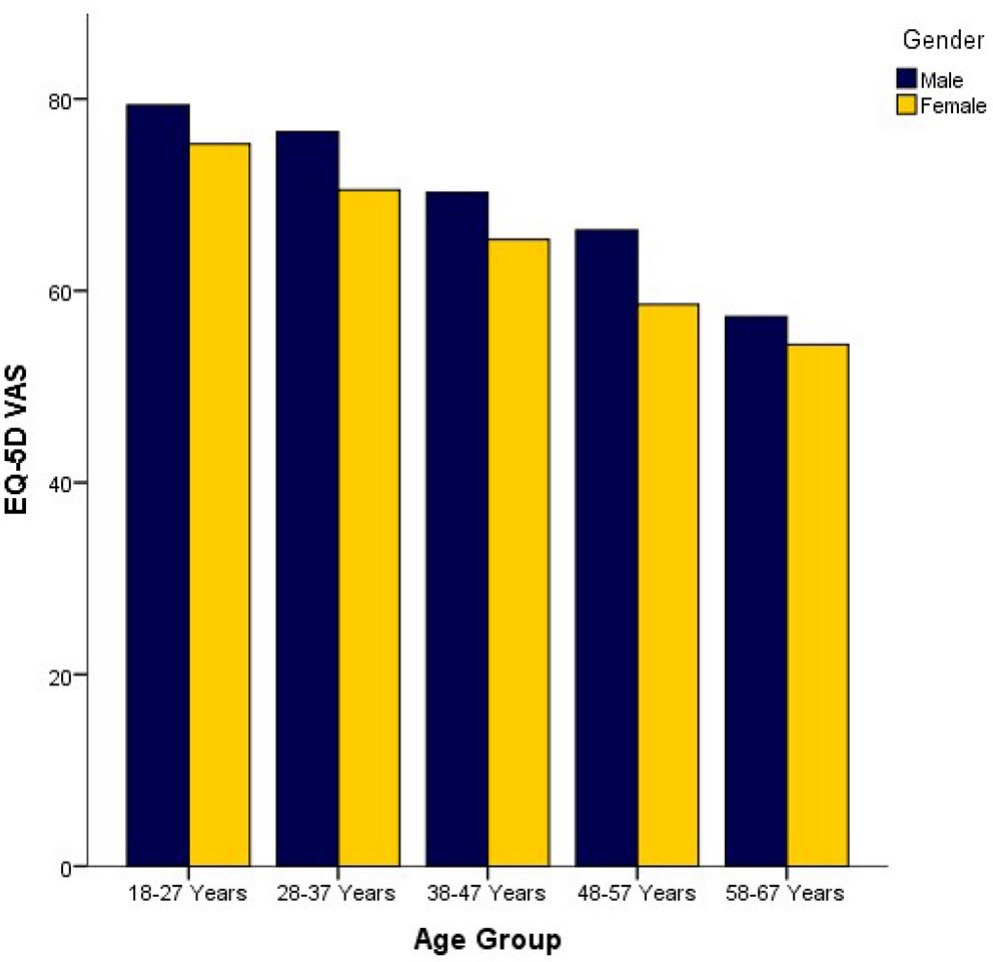
Mean E.Q. 5D VAS vs. Age Groups in terms of gender.

Mean E.Q. 5D VAS vs. age groups in terms of gender shown in Figure 1 represent the mean EQ-VAS ratings vs. age groups in terms of gender. Mean EQ-VAS decline with increasing age. Similarly, males of all ages stated higher EQ-VAS ratings than females.

### Comparison of Demographics Characteristics Among Mean EQ5D Index and VAS Score

Comparison of Demographics characteristics and Mean EQ5D Index Score shown n Table 5 which showed association among demographics and EQ-5D score, it was found that all demographics age (*p* < 0.001), gender (*p* < 0.001), marital status (*p* < 0.001), education (*p* < 0.001), Occupation (*p* < 0.001), House Occupancy (*p* < 0.001) and city (*p* < 0.001) were significantly associated with mean EQ5D score except for residence (*p* = 0.523).

**Table 5:** Comparison of mean EQ5D index and VAS score with demographics characteristics.

**Description**	**Frequency (*N* = 16672)**	**Mean** **EQ-5D score**	**P-Value**	**Mean** **VAS Score**	***P*-Value**
Age^*^ (29.22 +11.63)			**0.001**		**0.001**
18–27 Years 28–37 Years 38–47 Years 48–57 Years 58–67 Years	10,107 3,221 1,723 814 807	0.78777 ± 0.297666 0.75087 ± 0.289333 0.65817 ± 0.365273 0.62286 ± 0.351476 0.47192 ± 0.475093		0.78866 ± 0.243474 0.75075 ± 0.233192 0.67720 ± 0.264869 0.64438 ± 0.256544 0.54602 ± 0.319936	
Gender^**^			**0.001**		**0.001**
Male Female	9,014 7,658	0.75634 ± 0.325662 0.72928 ± 0.327667		0.76363 ± 0.258327 0.73619 ± 0.253819	
Marital status^*^			**0.001**		**0.001**
Single Married Widowed	9,849 6,643 180	0.78452 ± 0.303316 0.68877 ± 0.344542 0.57217 ± 0.487423		0.78655 ± 0.245407 0.70164 ± 0.260944 0.64061 ± 0.341055	
Education^*^			**0.001**		**0.001**
No Education Primary Middle Matric Intermediate Bachelors Graduate Masters Post Graduate (M.Phil./PhD)	900 324 443 1,782 3,621 4,629 1,881 2,909 183	0.56394 ± 0.439869 0.51837 ± 0.469135 0.59835 ± 0.439314 0.73163 ± 0.304067 0.77660 ± 0.282160 0.77089 ± 0.301769 0.77687 ± 0.286559 0.74292 ± 0.340580 0.84777 ± 0.315588		0.61903 ± 0.304270 0.57494 ± 0.309120 0.63865 ± 0.310485 0.73852 ± 0.245105 0.77451 ± 0.236791 0.77328 ± 0.244985 0.77191 ± 0.231265 0.75205 ± 0.264250 0.77191 ± 0.250585	
Occupation^*^			**0.001**		**0.001**
Student Own Business Government Job Private Job Engineer Doctor Labor House Wife Jobless None Teacher	2,643 1,169 2,111 3,088 484 652 543 1,033 1,214 1,959 1,776	0.84088 ± 0.224002 0.72573 ± 0.346666 0.77424 ± 0.309014 0.74876 ± 0.306931 0.79890 ± 0.305008 0.76781 ± 0.305614 0.62058 ± 0.378497 0.59195 ± 0.393424 0.70445 ± 0.358444 0.65072 ± 0.409030 0.74876 ± 0.321729		0.83135 ± 0.203311 0.73879 ± 0.267878 0.77626 ± 0.244467 0.75241 ± 0.249228 0.80788 ± 0.260604 0.76670 ± 0.235966 0.65657 ± 0.284988 0.62466 ± 0.269849 0.71723 ± 0.267481 0.68457 ± 0.301647 0.74979 ± 0.251808	
Income^*^			**0.001**		**0.001**
No Income 1–5,000 5,001–10,000 10,001–20,000 20,001–30,000 30,001–40,000 40,001–50,000 More than 50,000 Not Disclosed	4,418 961 819 2,706 3,057 1,120 773 700 2,118	0.74410 ± 0.337316 0.81052 ± 0.279585 0.71783 ± 0.312019 0.76322 ± 0.304374 0.74327 ± 0.342747 0.69776 ± 0.362483 0.72316 ± 0.300875 0.78609 ± 0.301450 0.71764 ± 0.324165		0.75463 ± 0.258420 0.80714 ± 0.242742 0.72483 ± 0.237883 0.76608 ± 0.245633 0.75214 ± 0.264118 0.71940 ± 0.286474 0.72435 ± 0.248859 0.78651 ± 0.233167 0.72207 ± 0.254781	
Residence^**^			0.523		0.175
Urban Rural	14,262 2,410	0.74146 ± 0.331710 0.75841 ± 0.296139		0.74999 ± 0.259901 0.75713 ± 0.236252	
House Occupancy^**^			**0.001**		**0.001**
Own Rent	13,484 3,188	0.76308 ± 0.306714 0.66281 ± 0.390784		0.76525 ± 0.245768 0.69086 ± 0.290640	
City^*^			**0.001**		**0.001**
Islamabad Rawalpindi Karachi Hyderabad	1,313 1,269 1,215 1,248	0.58449 ± 0.457091 0.58533 ± 0.452186 0.78845 ± 0.284156 0.79255 ± 0.299152		0.63919 ± 0.338478 0.638 ± 0.334693 0.78838 ± 0.237154 0.79792 ± 0.242552	
Sukkur Quetta Sargodha Peshawar Lahore Faisalabad Loralai Sawat Multan Sibi A.J.K. Gilgit Baltistan	606 1,507 1,330 1,237 1,270 1,232 1,187 734 408 1,198 445 473	0.86836 ± 0.165347 0.71596 ± 0.314889 0.65882 ± 0.378149 0.79864 ± 0.240400 0.77402 ± 0.228286 0.70520 ± 0.343909 0.84603 ± 0.266478 0.80527 ± 0.184589 0.82385 ± 0.213305 0.86967 ± 0.242968 0.69102 ± 0.282221 0.72556 ± 0.359531		0.85045 ± 0.162068 0.72378 ± 0.238152 0.67674 ± 0.279798 0.78614 ± 0.203089 0.76041 ± 0.197645 0.71452 ± 0.265283 0.83796 ± 0.187988 0.79437 ± 0.214297 0.82913 ± 0.232448 0.85327 ± 0.172969 0.69094 ± 0.216751 0.73615 ± 0.273247	
**Total**	**16672**	**0.74391** **±0.326853**		**0.75103** **±0.256623**	

* *Kruskal Wallis Test*.

** *Mann Whitney Test*.

***Sig. level 0.05***.

A comparison of Demographics characteristics and VAS Score is shown in Table 5 showed an association between demographics and VAS score. It was found that all demographics age (*p* < 0.001), gender (*p* < 0.001), marital status (*p* < 0.001), education (*p* < 0.001), Occupation (*p* < 0.001), House Occupancy (*p* < 0.001) and city (*p* < 0.001) were significantly associated with mean VAS score except for residence (*p* = 0.175).

### Associations Between Demographic Characteristics and Health Problems Reported in Five Dimensions

Binary logistic regression models showed in Table 6. Those with older age had significantly higher odds (Odds Ratio (OR) ranging from 1.1 to 7.4) of reporting health problems. Women were less likely (OR ranging from 1.3 to 1.4) than men to report problems in mobility, pain/discomfort, and anxiety/depression.

**Table 6:** Associations between demographics and health problems reported in five dimensions.

	**Mobility** **OR (95% CI)**	**Self-Care** **OR (95% CI)**	**Usual activities** **OR (95% CI)**	**Pain and Discomfort** **OR (95% CI)**	**Anxiety and Depression** **OR (95% CI)**
**Age Group**					
18–27 Years 28–37 Years 38–47 Years 48–57 Years 58–67 Years	Ref 1.3 (1.2–1.4) 2.8 (2.5–3.1) ^**^ 2.4 (2.0–2.7) ^**^ 7.4 (6.3–8.6) ^**^	Ref 0.89 (0.77–1.01) 0.93 (0.78 – 1.10) 1.12 (0.91–1.38) 4.08 (3.35–4.97) ^**^	Ref 1.24 (1.12–1.37) ^**^ 1.89 (1.66–2.15) ^**^ 1.10 (0.94–1.30) 3.56 (2.95–4.25) ^**^	Ref 1.54 (1.39–1.70) ^**^ 2.46 (2.16–2.79) ^**^ 2.67 (2.26–3.16) ^**^ 5.38 (4.43–6.52) ^**^	Ref 1.28 (1.16–1.41) ^**^ 1.66 (1.46–1.88) ^**^ 1.45 (1.20–1.71) ^**^ 1.14 (0.96–1.36)
**Gender**					
Male Female	Ref 1.3 (1.2–1.4) ^**^	Ref 0.93 (0.84–1.03)	Ref 0.91 (0.84–0.98)	Ref 1.41 (1.31–1.52) ^*^	Ref 1.20 (1.11–1.29) ^**^
**Marital Status**					
Single Married Widowed	Ref 2.2 (2.1–2.4) ^**^ 2.0 (1.5–2.8)	Ref 1.21 (1.08–1.36) ^**^ 1.10 (0.75–1.61)	Ref 1.03 (0.94–1.12) 1.85 (1.36–2.52) ^**^	Ref 1.20 (1.10–1.32) ^**^ 2.18 (1.56–3.06) ^**^	Ref 1.40 (1.29–1.52) ^**^ 1.76 (1.29–2.40) ^**^
**Education**					
None Primary Matric Intermediate Bachelors Masters Doctorate Middle Graduate	Ref 1.211 (0.94–1.56) ^*^ 0.49 (0.42–0.58) 0.36 (0.31–0.42) 0.33 (0.29–0.39) 0.38 (0.32–0.44) 0.41 (0.28–0.58) 0.76 (0.60–0.96) 0.28 (0.24–0.33)	Ref 2.48 (1.85–3.32) ^**^ 0.99 (0.79–1.23) 1.12 (0.91–1.38) 0.94 (0.76–1.16) 1.03 (0.82–1.29) 1.15 (0.64–2.06) 4.13 (3.16–5.39) ^**^ 1.15 (0.90–1.48)	Ref 1.93 (1.47–2.53) ^**^ 1.05 (0.88–1.25) 0.75 (0.63–0.89) ^**^ 0.82 (0.69–0.97) 0.71 (0.59–0.85) ^**^ 0.31 (0.20–0.49) ^**^ 1.34 (1.05–1.71) 0.86 (0.70–1.05)	Ref 0.91 (0.68–1.22) 0.49 (0.41–0.60) ^**^ 0.59 (0.49–0.70) ^**^ 0.51 (0.43–0.61) ^**^ 0.60 (0.50–0.73) ^**^ 0.11 (0.07–0.19) ^**^ 0.51 (0.39–0.66) ^**^ 0.57 (0.46–0.70) ^**^	Ref 3.08 (2.31–4.11) ^**^ 0.82 (0.82–1.17) 1.23 (1.23–1.72) ^**^ 0.98 (0.98–1.38) 1.02 (1.02–1.46) 0.21 (0.21–0.53) ^**^ 0.68 (0.68–1.11) 1.24 (1.24–1.84) ^**^
**Occupation**					
Student Own Business Government Job Private Job Engineer Doctor Labor House Wife Jobless None Teacher	Ref 2.61 (2.2–3.0) ^*^ 1.70 (1.4–1.9) ^*^ 1.78 (1.5–2.0) 1.84 (1.4–2.3) 1.70 (1.3–2.1) 3.27 (2.6–4.0) ^*^ 6.07 (5.1–7.1) ^**^ 2.13 (1.8–2.5) ^**^ 2.99 (2.5–3.5) ^**^ 1.80 (1.5–2.0)	Ref 1.33 (1.10–1.73) 0.58 (0.41–0.73) ^**^ 0.93 (0.74–1.18) 0.88 (0.63–1.23) 0.33 (0.22–0.49) ^**^ 1.59 (1.16–2.19) ^*^ 2.34 (1.18–2.95) ^**^ 2.47 (2.01–3.02) ^**^ 1.95 (1.58–2.41) ^**^ 1.06 (0.81–1.32)	Ref 1.16 (0.94–1.44) 0.98 (0.80–1.21) 1.04 (0.86–1.25) 0.54 (0.41–0.72) ^**^ 0.72 (0.56–0.94) 1.53 (1.18–1.99) ^**^ 1.50 (1.24–1.80) ^**^ 2.46 (2.11–2.88) ^**^ 1.55 (1.31–1.84) ^**^ 1.18 (0.95–1.45)	Ref 2.53 (2.04–3.14) ^**^ 1.23 (0.99–1.52) 1.97 (1.62–2.38) ^**^ 0.80 (0.60–1.07) 1.92 (1.49–2.46) ^**^ 2.79 (2.14–3.64) ^**^ 2.06 (1.71–2.48) ^**^ 2.31 (1.99–2.68) ^**^ 1.51 (1.27–1.78) ^**^ 1.52 (1.22–1.89) ^**^	Ref 1.97 (1.6–2.43) ^**^ 1.68 (1.37–2.05) ^**^ 1.61 (1.34–1.94) ^**^ 0.69 (0.52–0.9) ^*^ 1.70 (1.33–2.17) ^**^ 1.99 (1.53–2.57) ^**^ 1.54 (1.29–1.83) ^**^ 1.86 (1.61–2.15) ^**^ 1.33 (1.14–1.56) ^**^ 1.59 (1.3–1.95) ^**^
**Income** ^**a**^					
No Income 1–5,000 5,001–10,000 10,001–20,000 20,001–30,000 30,001–40,000 40,001–50,000 More than 50,000 Not Disclosed	Ref 1.10 (0.93–1.3) ^*^ 0.82 (0.7–0.9) ^**^ 1.13 (1.0–1.2) ^**^ 1.11 (0.9–1.2) ^**^ 1.78 (1.5–2.1) ^**^ (0.8–1.2) 0.81 (0.6–0.9) ^*^ 1.20 (1.0–1.3) ^**^	Ref 1.30 (0.97–1.74) 1.69 (1.33–2.15) ^**^ 2.51 (1.98–3.17) ^**^ 3.54 (2.72–4.61) ^**^ 4.07 (3.07–5.39) ^**^ 0.68 (0.45–1.03) 1.70 (1.29–2.24) ^**^ 2.94 (2.51–3.44) ^**^	Ref 1.60 (1.28–2.00) ^**^ 1.21 (1.00–1.48) 1.75 (1.45–2.12) ^**^ 1.59 (1.28–1.98) ^**^ 2.95 (2.33–3.72) ^**^ 1.57 (1.23–2.03) ^**^ 1.13 (0.90–1.41) 2.00 (1.75–2.28) ^**^	Ref 0.85 (0.68–1.08) 0.77 (0.63–0.94) 0.65 (0.53–0.78) ^**^ 0.85 (0.69–1.07) 0.85 (0.67–1.08) 0.51 (0.44–0.66) ^**^ 0.44 (0.35–0.55) ^**^ 1.12 (0.97–1.28)	Ref 1.08 (0.87–1.34) 0.70 (0.58–0.85) ^**^ 0.75 (0.62–0.91) ^*^ 0.60 (0.48–0.74) ^**^ 0.75 (0.59–0.94) 0.29 (0.23–0.38) ^**^ 0.44 (0.35–0.55) ^**^ 1.30 (1.14–1.47) ^**^
**Residence**					
Urban Rural	Ref 1.36 (1.2–1.4)	Ref 0.68 (0.59–0.78)^**^	Ref 0.84 (0.76–0.93) ^**^	Ref 0.92 (0.84–1.02)	Ref 0.66 (0.60–0.73) ^**^
**House Occupancy**					
Own Rent	Ref 1.61 (1.4–1.7) ^**^	Ref 1.74 (1.57–1.93) ^**^	Ref 1.12 (1.12–1.33) ^**^	Ref 1.31 (1.21–1.43) ^**^	Ref 1.42 (1.31–1.54) ^**^
**Cox and Snell R2**	0.098	0.081	0.068	0.116	0.064
**Nagelkerke R2**	0.143	0.137	0.094	0.156	0.085

a *Income in PKR; 1 PKR =0.0095 US Dollar*.

* *p < 0.05*.

** *p < 0.01*.

*OR, odds ratio; CI, confidence interval*.

After adjustment of demographics, lifestyle and socioeconomic factors remained significantly associated with self-reported health problems in EQ-5D-3L. Rural residents had odds of reporting problems in self-care (OR = 0.6), usual activities (OR = 0.8) and anxiety/depression (OR=0.6). However, people who live in rental houses had slightly higher odds of reporting problems in all five domains (OR ranging from 1.1 to 1.7). The odds of reporting health problems decreased with educational achievement. Those who graduated having an OR of 0.2–1.2 compared with those with less than primary school education and middle education had higher odds.

People who have no job had higher odds of reporting health problems (1.3–2.9) than their employed counterparts. Surprisingly, housewives showed a higher likelihood of reporting health problems in all five health dimensions (OR ranging from 1.5 to 6.0). The widowed showed higher odds of reporting problems in usual activities, pain/discomfort, and anxiety/depression (OR ranging from 1.7 to 2.1). People living married tend to report slightly higher odds ratios in reporting problems in mobility, self-care, pain/discomfort, and anxiety/depression (OR ranging from 1.0 to 2.2). Respondents who earn between 30 and 40 k had higher (OR = 4.0) in self-care, Usual activities (OR = 2.9), and mobility (OR = 1.78).

The logistic regression models also confirmed associations between self-reported health problems and younger age. For example, those aged 28–37 years had lower odds (OR = 1.2–1.5) of reporting health problems in usual activities, pain/discomfort, and anxiety/depression. Similarly, respondents who earn more than 50 k had lower odds (OR = 0.8–1.7).

### Associations Among Demographics With an EQ5D Index Score

The model explains 7% (Nagelkerke's R^2^ = 0.072) of the variance of the dependent variable. That means a suboptimal value, perhaps because the covariates included are structural, and HRQOL is subjective. The regression model showed that age was the best predictor of the EQ-5D index after adjusting for the rest of the covariates (beta = 0.19; *p* < 0.001). House occupancy was the second-best predictor of the EQ-5D index after adjusting for the rest of the covariates (beta = 0.11; *p* < 0.001), as shown in Table 7.

**Table 7:** Associations among EQ5D Index score and demographics.

**Model**	**Standardized Coefficients (Beta)**	**t**	**Sig**.
(Constant) Age group Gender Income Education Occupation Residence Marital status House occupancy	**0.188** 0.020 0.011 0.032 0.083 0.038 0.025 **0.108**	55.678 −21.055 −2.608 1.385 4.050 −10.715 5.067 −2.731 −14.412	0.000 **0.000** 0.009 0.166 0.000 0.000 0.000 0.006 **0.000**

*R Square = 0.072*.

*Adjusted R Square = 0.072*.

## Discussion

This study provides the health status of a population sample from Pakistan using the EQ-5D-3L tool. The values will be valuable as population norms to support the evaluation of health care in Pakistan. There are no other reported population norms compared with the present study. It is the first of its kind in Pakistan, where the evaluation of the EQ-5D-3L tool is used to measure the health status of the Pakistani people.

These norms can be used as standards when relating or comparing different groups' health status, i.e., disease state Q.O.L. with the general people in Pakistan. Unfortunately, there are no other reported population norms to be compared with the present study in Pakistan. However, various researchers measured patient-reported outcomes from time to time to investigate health-related quality of life in disease conditions (10, 32, 33).

The proportion of respondents who described total health was 37.6% in this study. However, almost half of the observations reported sixty-five percent of a European six countries population who did not specify any problems in the EQ-5D-3L dimensions (34). Similarly, full health in Sri Lanka was seen in 60.7% population, which is again half of this study's findings (24). However, a study conducted in Sweden to evaluate Swedish EQ-5D health states using overall population health survey data highlighted that full health was less than 50%, consistent with the present study's findings (35). The current study results showed the maximum proportion of the population reported full health, i.e., not having moderate or severe problems on any EQ-5D dimensions because the mainstream of the community was healthy; this is consistent with the findings of the study in China (27).

The present study showed the mean VAS value of Pakistan was (0.75), which is noticeably different from Sri Lanka (0.81) (24), Singapore (0.95) (36), U.K. (0.85) (23), and Sweden (0.87).

This study highlighted that with the increase in age, Mean VAS decreased, and moderate and severe problems were frequently reported with increase in age; older people had a higher occurrence of problems in all EQ-5D-3L dimensions and lesser EQ-VAS scores as compared to younger age and highlighted decreased health status with age these findings are consistent with health measure of Brazilian adult population (22) and population health status in China (27). Respondents who reported moderate and severe problems in the present study are high in elderly age, particularly pain or discomfort and depression or anxiety, which is in line with the survey conducted in the U.K., where they highlighted extreme problems with mobility and self-care hardly stated. However, a maximum of stated problems with pain or discomfort was seen (6). Results showed that age groups remained significant after controlling for other socio-demographic factors in logistic regression analysis. These outcomes remained parallel with findings in Singapore (37) and the U.K. (23).

Concerning differences between genders, In the present study, it is reported that females have additional health problems and reported a minimum of complete health, health status is low or decreased among women as compared to men of all age groups, which is consistent or similar to studies reported more inferior health status than males, in China (27). Sri Lanka (24) and Singapore (34) which is identical with EQ-5D population health studies in other countries. Besides EQ-5D dimensions, it is also estimated that men have a better VAS score than females and have more health problems and a low ratio of complete health. The lack of health status among males and females in Pakistan is parallel to Australia (21), Singapore (37), and Sri Lanka (24). Furthermore, it is highlighted in the results that men described higher HRQOL than females in all age groups, corresponding findings seen in Denmark (26); similarly, it is shown that females stated inferior health status than males in China and other countries (27, 34, 38). Furthermore, T.T.O. and VAS values reported being significantly related to gender (*p* < 0.001), which is in line with a study in Sri Lanka (24) and contrary to findings in Sweden (35).

The present study showed that income was not a significant factor. Though self-reported income monthly or yearly data should be measured carefully, people might have misjudged answers as most respondents worked but preferred not to disclose their income. This is consistent with a study where the author highlighted household income, not a significant aspect of the Sri Lankan population (24). Though we tried to include respondents from Rural areas as well to know about their health-related quality of life, however, their ratio was small because major participants were from cities of Pakistan; therefore, the amount of income they produce is not highlighted on significant population because it is known fact Pakistan being agriculture country generates its wealth from agriculture and this agriculture mainly comes from rural areas this is consistent with findings in Sri Lanka (24). Though, in some countries, household income is a good indicator of better health status (24, 25, 37).

In Pakistan, people with education or higher education tend to have better health and are less likely to report moderate and severe problems, in contrast to those who have no or low-level education have reported some and extreme problems, and this is similar to the condition in high-income nations (6) and Asian country Sri Lanka (24). Educated individuals are less likely to progress disease, a particularly chronic one. They have better health and a low chance of getting diseases than disadvantaged people who are less likely to adjust lifestyle variation and precautionary actions, resulting in an enhancement of HRQOL (10).

The present study highlighted Pakistani EQ-5D-3L based utility weights to compute the Pakistani population sample's health status. It would be an asset to study. This might be claimed to be a valid method than using alternative or other country's utility weights to compute the health-related quality of life population norms (24, 37). The present study showed significant differences among the EQ-5D-3L VAS values and values produced from utility weight for the participants' health states (24, 28).

## Conclusion

The current study concluded that health status population norms can help to make the health status of Pakistan's general persons. Standards and values computed in this study of Pakistanis show a decrease with age; female gender had poor health in all age groups than males. Socioeconomically deprived groups have inferior health status than more advantaged. The trends detected in high-income nations were usually similar to Pakistan. These standard values allow healthcare providers and researchers to improve deprived groups' health status and associate the health status of diseased conditions with these Pakistani norms. The authors strongly recommend using these norms in health policy and their induction in the census to target each district or city to explore more findings.

It is shown in the current finding that the regression model reported demographics, which included Age, City, Gender, Education, Occupation, Residence, and House occupancy being significantly associated with HRQOL. However, house occupancy and age were rated as predictors of HRQOL in the current cohort.

## Recommendations

This unique and novel study provided Pakistani population norm data available so far. These can be utilized in policy-making and decisions or verdicts while making and devising health policy for Pakistan. From this information, it is suggested to include or add an EQ-5D-3L survey in the national population census and demographic and health survey to know about Pakistan's health status and health status variations among various demographics. Policymakers and government officials of Pakistan can commence such data assortment. Involvement gained in the present analysis can assess and evaluate the better health-related quality of life information for Pakistan in forthcoming. Researchers also recommend that researchers estimate the health-related quality of life in disease states or patient-reported outcomes to measure their outcomes with Pakistani population norms rather than measure with other country profiles. Furthermore, improving or enhancing decreasing age Q.O.L. predictors is recommended to cope with chronic diseases or improve health later.

## Data Availability Statement

The raw data supporting the conclusions of this article will be made available by the authors, without undue reservation.

## Ethics Statement

The studies involving human participants were reviewed and approved by Faculty of Pharmacy and Health Sciences University of Baluchistan Quetta. The patients/participants provided their written informed consent to participate in this study.

## Author Contributions

AN and SR contributed to the design and conception of the study. FK, MT, and MFS organized and prepared data file. AN performed statistical analysis. KM, RY, BT, and MS wrote the sections of manuscript. AN and SK wrote the first draft of the manuscript. NH supervised the study. FK, MFS, BT, KM, SK, and MD co-supervised the research, data collection, and completed required ethical requirements from Khyber Pakhtunkhwa, Islamabad, Balochistan, AJ&K, and Sindh respectively. NH, AN, and MD commented on the manuscript. All authors contributed in manuscript revision, proofread, and approved the submitted version.

## Conflict of Interest

The authors declare that the research was conducted in the absence of any commercial or financial relationships that could be construed as a potential conflict of interest.

## Publisher's Note

All claims expressed in this article are solely those of the authors and do not necessarily represent those of their affiliated organizations, or those of the publisher, the editors and the reviewers. Any product that may be evaluated in this article, or claim that may be made by its manufacturer, is not guaranteed or endorsed by the publisher.
